# Alteration in molecular properties during establishment and passaging of endometrial carcinoma patient-derived xenografts

**DOI:** 10.1038/s41598-023-35703-6

**Published:** 2023-05-25

**Authors:** Toshio Imai, Hiroshi Yoshida, Yukino Machida, Mizuki Kuramochi, Hitoshi Ichikawa, Takashi Kubo, Mami Takahashi, Tomoyasu Kato

**Affiliations:** 1 Central Animal Division, National Cancer Center Research Institute, 5-1-1 Tsukiji, Chuo-ku, Tokyo, 104-0045 Japan; 2 Department of Diagnostic Pathology, National Cancer Center Hospital, Tokyo, Japan; 3 Department of Clinical Genomics, National Cancer Center Research Institute, Tokyo, Japan; 4 Department of Gynecology, National Cancer Center Hospital, Tokyo, Japan

**Keywords:** Cancer, Drug discovery, Oncology

## Abstract

Patient-derived xenograft (PDX) tumor models are known to maintain the genomic and phenotypic profiles, including the histopathological structures, of the parental tumors. On the other hand, unique enrichment of single-nucleotide variants or copy number aberrations has been reported in several types of tumors. However, an understanding of endometrial carcinoma PDXs is limited. The purpose of the present study was to clarify the presence or absence of the molecular properties of endometrial carcinomas in PDXs passaged up to eight times. Established PDXs of endometrioid carcinomas maintained their histopathological characteristics, but those of carcinosarcomas predominantly consisted of sarcomatous components when compared to the parental tumors. Alterations in the proportion of cells with positive/negative immunohistochemical staining for estrogen receptor, PTEN, PAX8, and PAX2 were observed, whereas the proportions of cells with AE1/AE3, TP53, ARID1A, PMS2, and MSH6 staining were unchanged. Variants of cancer-associated genes were compared between PDXs and parental tumors. Mutations in *POLE* and a frameshift deletion in *BRCA1* were observed in the parental tumor tissue in each of the six cases, and additional genomic alterations, which were not apparently related to histopathological and immunohistochemical alterations, were found in the PDXs of these cases. The genomic and phenotypic alterations observed between endometrial carcinoma PDXs and parental tumors were partly associated with endometrial cancer-specific characteristics related to cellular differentiation and gene mutations.

## Introduction

Cancer cell/tissue transplantation animal models have been utilized for the evaluation of anticancer drugs and for basic cancer research. Transplantation of immortalized cell lines into immunodeficient mice, such as athymic nude mice, has been the most commonly used method based on the advantage of good reproducibility in terms of growth rate and phenotype in each laboratory. However, immortalized cell lines have been adapted to grow indefinitely in artificial culture conditions that often differ between laboratories, resulting in minimal resemblance to the parental tumors and substantial limitations for estimating the preclinical efficacy of drugs^1,2^. In addition, the selection of genotype/phenotype-matched patients in clinical trials, as well as corresponding in vitro/in vivo preclinical models, has become crucial for the development of molecular targeted drugs^3,4^.

Patient-derived xenograft (PDX) models are generated by engraftment of immunodeficient mice with patients’ primary tumor cells or tissues. Over the past several decades, immunodeficient mouse models have continuously evolved from T-cell deficient athymic nude mice to severe combined immunodeficient (SCID) mice lacking both functional T and B lymphocytes^5^. A spontaneously developing nonobese diabetic mouse model that displayed mononuclear infiltration in and around pancreatic islets, as well as simultaneously decreased NK-cell activity and macrophage/dendritic cell dysfunction was reported later by Kikutani and Makino^6^, and this followed by the development of nonobese diabetic-severe combined immuno-deficiency (NOD-SCID) mice^7,8^ and, eventually, genetically modified IL-2 receptor γ-deficient mice, e.g., NOD/Shi-scid/IL-2RγKO [NOG] mice^9^ and NOD/LtSz-scid/IL-2Rγ null [NSG] mice^8^. As a result of the stepwise development of immunodeficient mouse models, engraftment rates of PDXs have improved^10–12^, and genomic and gene expression/immunohistochemical profiles and histopathological structures of parental tumors are often maintained in PDXs in severely immunodeficient NOD-SCID and NOG/NSG mice^11–14^. Therefore, a large-scale PDX library using severely immunodeficient mice has been developed worldwide for drug screening and preclinical approaches for personalized medicine^15–17^.

Endometrial cancer is the sixth most commonly diagnosed cancer worldwide in women, with 417,000 new cases and 97,000 deaths in 2020^18^. PDX models derived from endometrial cancer samples have been histopathologically and molecularly characterized and demonstrated to retain patient tumor characteristics as an individualized approach to evaluate the efficacy of novel therapies and to identify treatment-response biomarkers^19,20^. However, whole-genome sequencing for breast cancer patient-derived xenografts in NOD-SCID mice revealed some enrichment of unique single-nucleotide variants not present in primary tumors; the genome was relatively stable without significant accumulation of DNA structural rearrangements in the PDX. Therefore, this enrichment was suggested to be a result of adaptation to transplantation into the new microenvironment, but the single-nucleotide variants could also have been present in the parental tumor below detectable limits^21^. Another study showed that many copy number aberrations found in sarcoma PDXs in athymic nude mice were frequently observed in patients with advanced sarcoma, suggesting that xenografts may represent the genomic rearrangement intrinsic to tumor progression in some way^22^. Recently, primary acute myeloid leukemia (AML) cells, including potential chemotherapy-resistant clones, showed a high engraftment efficiency in AML-PDX models, and these cells enriched the preexisting treatment-resistant subclone population^23^. The genotypic conversion that can occur over multiple passages in certain types of tumor-derived xenografts should be investigated in detail in major cancer types to clarify whether the genomic and phenotypic characteristics of the parental tumors are maintained in PDX models developed using severely immunodeficient mice. For endometrial cancer, molecular and histological features of parental tumors have been reported to be retained in PDXs^24,25^. However, in a recent study by Bonazzi et al., whole-genome sequencing was performed on endometrial cancer PDXs with four molecular subtypes, (e.g., *POLE* mutations, mismatch-repair deficiency (MMRd), copy number high/*TP53* mutations, and no specific molecular profile). They found that mutational heterogeneity was more frequent in MMRd PDXs than in other PDXs^26^.

The purpose of the present study was to clarify the presence or absence of phenotypic alterations with up to eight passages in endometrial carcinoma patient-derived xenografts using NOG/NSG mice. To the best of our knowledge, information on changes in specific maker proteins between endometrial cancers and PDXs is limited^25^. Thus, phenotypic changes with establishment and passaging were first determined through histopathological and immunohistochemical testing of the parental tumors and PDXs. Second, targeted genomic sequence analysis of the parental tumor tissues and PDXs was performed to assess genotypic changes, and a comprehensive evaluation of immunohistochemical and genomic characteristics was conducted. Furthermore, the efficacy of paclitaxel was compared between endometrial carcinoma PDXs at passage 3 and those at passage 8. The present study revealed several genomic and phenotypic alterations in endometrial carcinoma PDXs in relatively early passages, suggesting that verification of genomic and phenotypic similarities between primary tumors and PDXs is required when using endometrial carcinoma-PDXs for drug screening and preclinical studies.

## Results

### Characteristics of primary endometrial carcinomas

The patient and pathological characteristics of the 12 endometrial cancer cases are summarized in Table 1. Six and two of 12 cases were diagnosed as G1/2 and G3 endometrioid carcinomas, respectively; three cases were carcinosarcomas, and one was mixed cell carcinoma.

**Table 1:** Patient and pathological characteristics, and engraftment of PDX of 12 endometrial cancer cases.

Case	Age	Diagnosis	Tumor grade	TNM, FIGO2008	Extra uterine metastasis	Engraftment of PDX
UXE-001	90	Endometrioid carcinoma, G1	G1	pT1aN0, IA	N	Yes
UXE-002	43	Endometrioid carcinoma, G1	G1	pT3aN0, IIIA	N	No
UXE-003	36	Endometrioid carcinoma, G1	G1	pT2N0, II	N	No
UXE-004	50	Carcinosarcoma, heterologous	High	pT1aN2, IIIC2	N	Yes
UXE-005	77	Mixed cell carcinoma (endometrioid G2 and serous)	G2/high	pT1bN0, IB	N	Yes
UXE-006	67	Endometrioid carcinoma, G1	G1	pT1aN0, IA	N	No
UXE-007	48	Endometrioid carcinoma, G1	G1	pT1aN0, IA	N	No
UXE-008	70	Carcinosarcoma, homologous	High	pT2N0, II	N	Yes
UXE-009	46	Carcinosarcoma, heterologous	High	pT3aN2, IIIC2	Ovary, para-aortic node	Yes
UXE-010	52	Endometrioid carcinoma, G1	G1	pT1bN0, IB	N	Yes
UXE-011	64	Endometrioid carcinoma, G3	G3	pT1aN1, IIIC1	N	No
UXE-012	63	Endometrioid carcinoma, G3	G3	pT3aN1M1, IVB	Peritoneal dissemination	No

*G1* grade 1, *G3* grade 3, *high* high-grade, *N* none.

### Engraftment rates and growth of PDX

**Figure 1:**
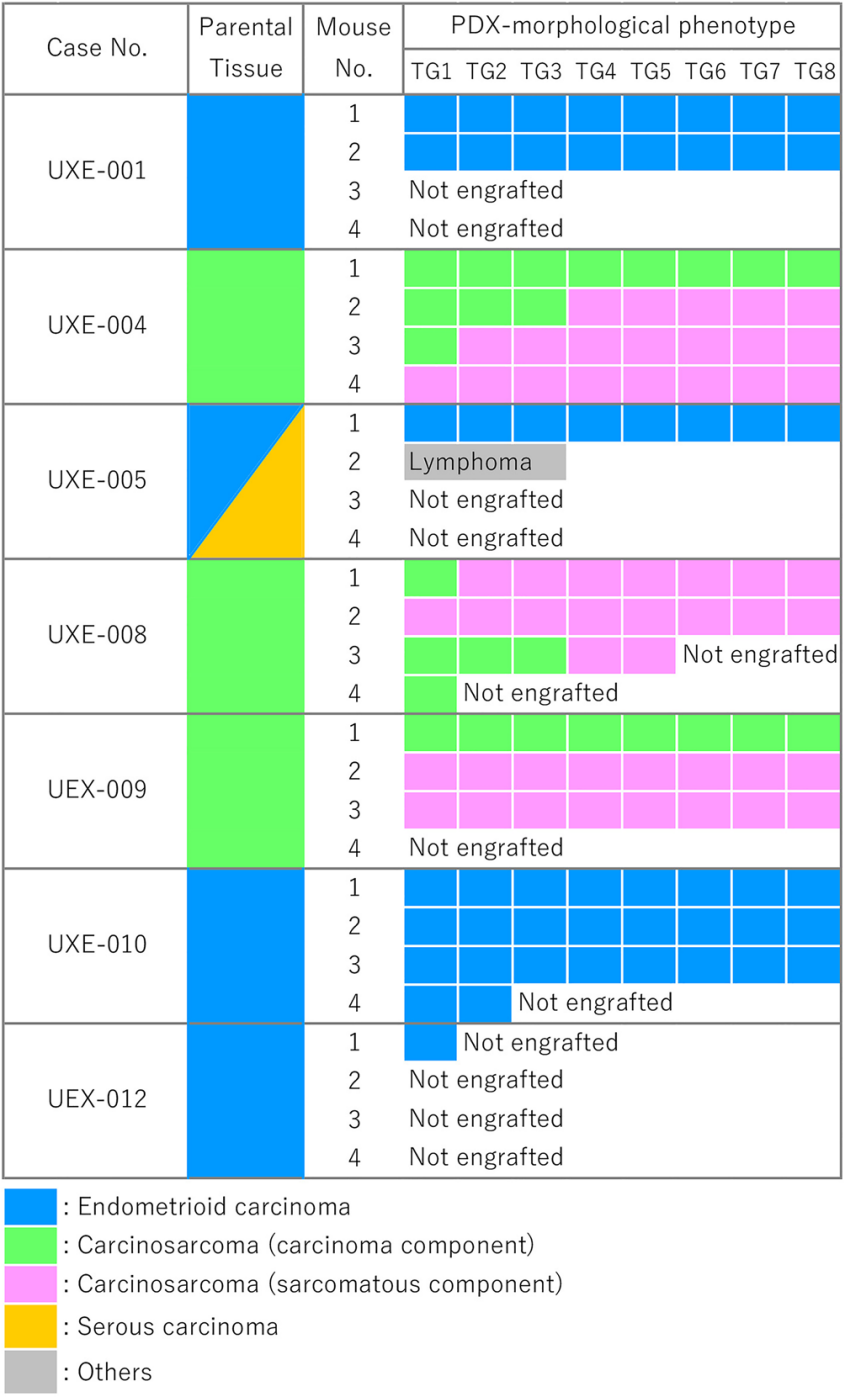
Histopathological phenotypes of PDXs from endometrial tumors. *PDX* patient-derived xenograft, *TG1-8* Trans Generation 1–8.

The success of engraftment of 12 endometrial carcinoma cases is illustrated in Fig. 1. Among endometrioid carcinoma tissues from nine cases (including one mixed carcinoma case), those from three cases (UXE-001, UXE-005, and UXE-010) showed successful engraftment and were passaged to the end of Trans Generation 8 (TG8) in 1 to 3 of 4 mice, and tissues from six cases were confirmed as not engrafted in all 4 mice. In contrast, among carcinosarcoma tissues from three cases (UXE-004, UXE-008, and UXE-009), all cases showed successful engraftment and were passaged to TG8 in 2 to 4 of 4 mice. The duration from implantation to passaging to the next generation, which could represent the growth of the PDX in each mouse, is summarized in Supplementary Fig. S1. The durations from implantation to passaging to the next generation in TG1 mice were longer than those in TG2 and later-passage mice in these cases of UXE-001, UXE-004, UXE-005, and UXE-009. However, a clear relationship between duration from implantation to passaging to the next generation and transplant generation was not observed in the cases of UXE-008 and UXE-010.

### Histopathology: the characteristics of endometrioid carcinoma were maintained, and the sarcomatous component showed predominance in carcinosarcomas

**Figure 2:**
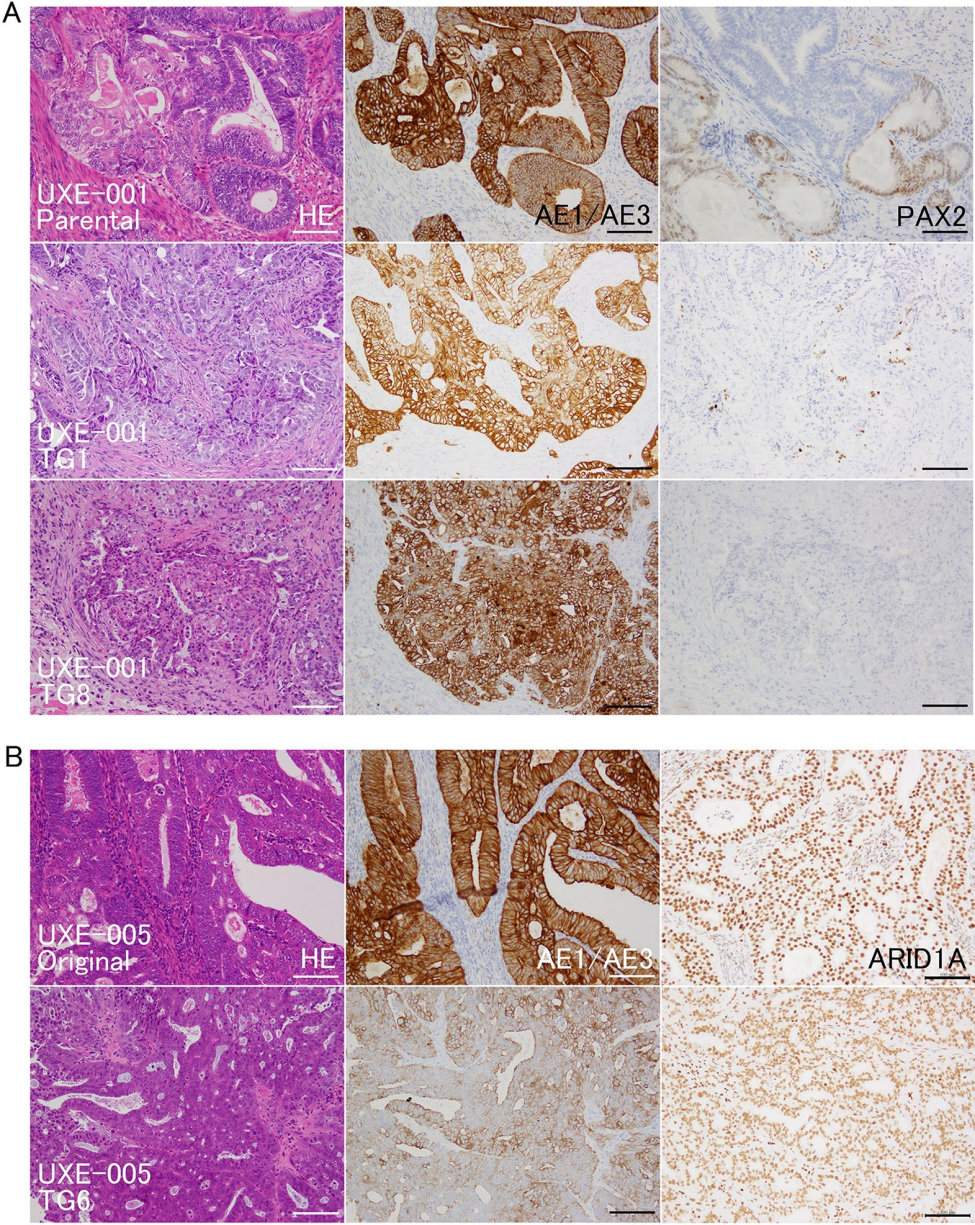

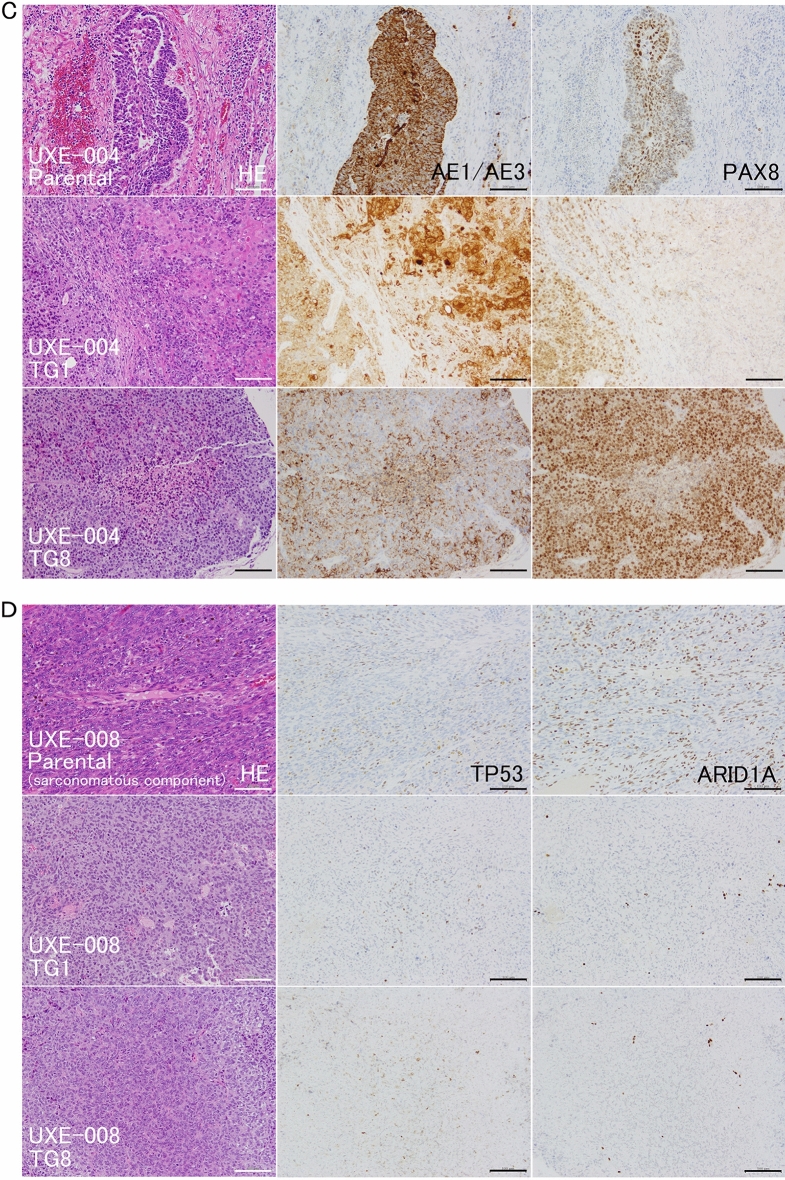
Histopathological and immunohistochemical findings of parental tumors and PDXs. (**A**,**B**) Left panels, HE staining. Middle and right panels present immunohistochemical staining of a serial section for each HE stained section on the left. HE; morphological characteristics, such as glandular structure and solid nests, are maintained in PDXs of endometrioid carcinoma cases (UXE-001 and UXE-005). (**C**,**D**) Sarcomatous components are prominent in PDXs of carcinosarcoma cases (UXE-004 and UXE-008). AE1/AE3; the positivity was almost maintained in PDXs (UXE-001, UXE-004, and UXE-005). PAX2; the proportions of positive cells gradually decreased in PDXs (UXE-001). ARID1A; the rate of positivity observed in the parental tumor tissues was almost maintained in the PDXs (UXE-005). PAX8; the proportions of positive cells gradually increased in PDXs (UXE-004). TP53 (wild-type pattern) and ARID1A (loss of expression); the staining patterns were almost maintained in PDXs (UXE-008). *HE* hematoxylin and eosin, *TG1, 6 and 8* TransGeneration 1, 6 and 8. Bar = 100 μm.

The morphological phenotypes of PDXs in each case are summarized in Fig. 1. Established PDXs of endometrioid carcinomas from two cases (UXE-001 and UXE-010) maintained their phenotypes from TG1 to TG8, with epithelial characteristics of glandular/papillary structures or solid growth (Fig. 2A, Supplementary Fig. S2A). For the PDXs of one case of mixed cell carcinoma with serous adenocarcinoma (UXE-005, Fig. 2B), the endometrioid carcinoma component with glandular/cribriform structures was predominant from TG1 to TG8. Tumor tissues of the mixed cell carcinoma transplanted to a mouse were replaced with lymphoma, which was indicated to be of human CD45-positive B-cell origin and frequently observed in PDXs of gastric and colorectal carcinomas^27,28^. Among PDXs of carcinosarcomas from three cases (UXE-004, UXE-008, and UXE-009), those implanted into two mice (one mouse each for two cases; UXE-004 and UXE-009) maintained their phenotype with epithelial characteristics; however, those implanted into other mice were predominantly composed of sarcomatous components from TG1 or later generations (Fig. 2C,D, Supplementary Fig. S3A).

### Immunohistochemistry: the proportions of ER-, PTEN-, PAX8-, and PAX-2-positive cells showed case-dependent alterations in PDXs

**Figure 3:**
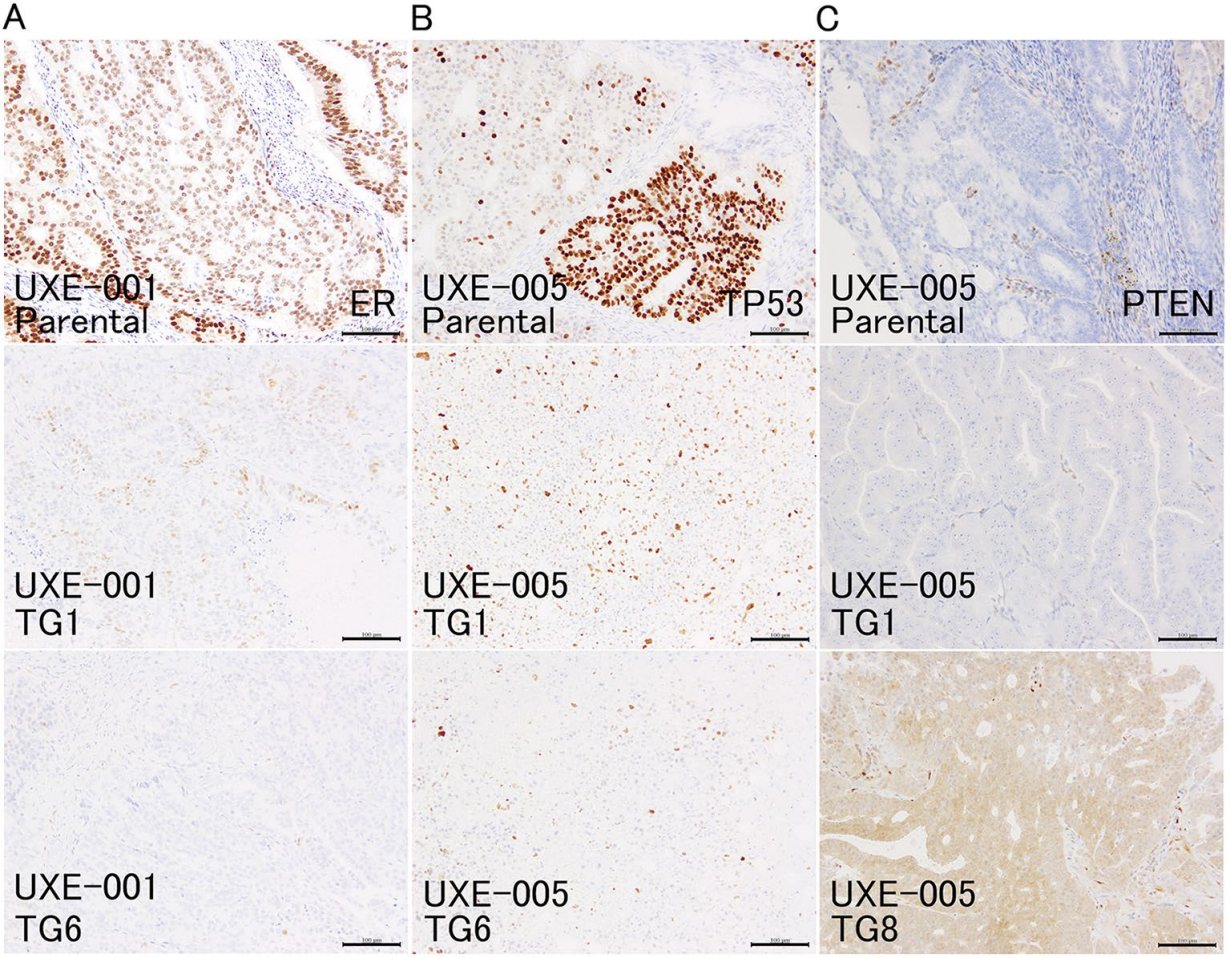
Immunohistochemical findings of parental tumors and PDX. (**A**) Immunohistochemical staining for ER. ER status switched from positive to negative in PDXs (UXE-001). (**B**) Immunohistochemical staining for TP53. TP53 staining was heterogeneous and partly positive (indicating a mutation in some cells), but PDXs showed wild-type expression patterns (UXE-005). (**C**) Immunohistochemical staining for PTEN. The proportions of PTEN-positive cells were increased in one case (UXE-005). *ER* estrogen receptor, *TG1 and 6* TransGeneration 1 and 6. Bar = 100 μm.

Immunohistochemistry (IHC), which has been applied for subclassification of endometrial cancer, as a surrogate marker for hormone sensitivity, was performed along with analysis of genetic and/or epigenetic alterations in TP53, PTEN, and ARID1A (which are frequent mutations in endometrial cancer) and DNA mismatch repair genes^29,30^. The overall results of IHC are summarized in Supplementary Table S1. An epithelial marker AE1/AE3^31^, which was positive not only in endometrioid carcinomas but also in epithelial components in carcinosarcomas, was still positive in almost all PDXs (Fig. 2A–C, Supplementary Figs. S2A and S3A). Estrogen receptor (ER), an endometrial cell marker, that suggests estrogen-dependent growth, was positive in all cases except one (UEX-009, Supplementary Fig. S3A). ER-positivity was maintained in PDXs in three cases (Supplementary Fig. S2A), and it became negative in two of five cases (UXE-001 and 008, Fig. 3A). TP53 showed scattered positivity (wild-type staining patterns) in the parental tumors, and this phenotype was maintained in PDXs from five of six cases (Fig. 2D, Supplementary Fig. S2B). In the parental tumor of one patient (UXE-005), TP53 staining was heterogeneous and partly positive (indicating a mutation in some cells), but PDXs showed wild-type expression patterns (Fig. 3B), suggesting that wild-type regions are overrepresented in engrafted PDXs. PTEN is a tyrosine phosphatase that negatively regulates the PI3K/AKT pathway^32^. The proportions of PTEN-positive cells were almost the same in one case (UXE-009, Supplementary Fig. S3B), and were increased in another case (UXE-005, Fig. 3C). A member of the pair-box (PAX) family of transcription factors PAX8^33^, a marker of Müllerian ducts^34^, was positive in endometrioid carcinomas (UXE-001 and -010), and this positivity was maintained in the PDXs (Supplementary Fig. S2C). On the other hand, PAX8 was mainly positive in epithelial components in parental tissues of carcinosarcomas, and the proportions of PAX8-positive cells were increased in the PDX for two cases (UXE-004 and -005, Fig. 2C) and negatively converted in the PDX for another case (UXE-008). PAX2 is a transcription factor that controls the development of the kidney, organs derived from the mesonephric (Wolffian) duct and those related to the Müllerian duct^35^. PAX2 appeared partly positive in three cases (UXE-001, UXE-008, and UXE-010) and gradually became negative with passages in the PDXs for all three cases PDXs (Fig. 2A, Supplementary Fig. S2C). For ARID1A (a subunit of SWI/SNF complex), which is immunohistochemically negative when it is mutated^36^, and the mismatch repair proteins PMS2 and MSH6, the rate of positivity observed in the parental tumor tissues was almost maintained in the PDXs (Fig. 2B, Supplementary Figs. S2B, S2C, S3B, and S3C).

### Mutation status was partially altered by PDX establishment and passaging

**Figure 4:**
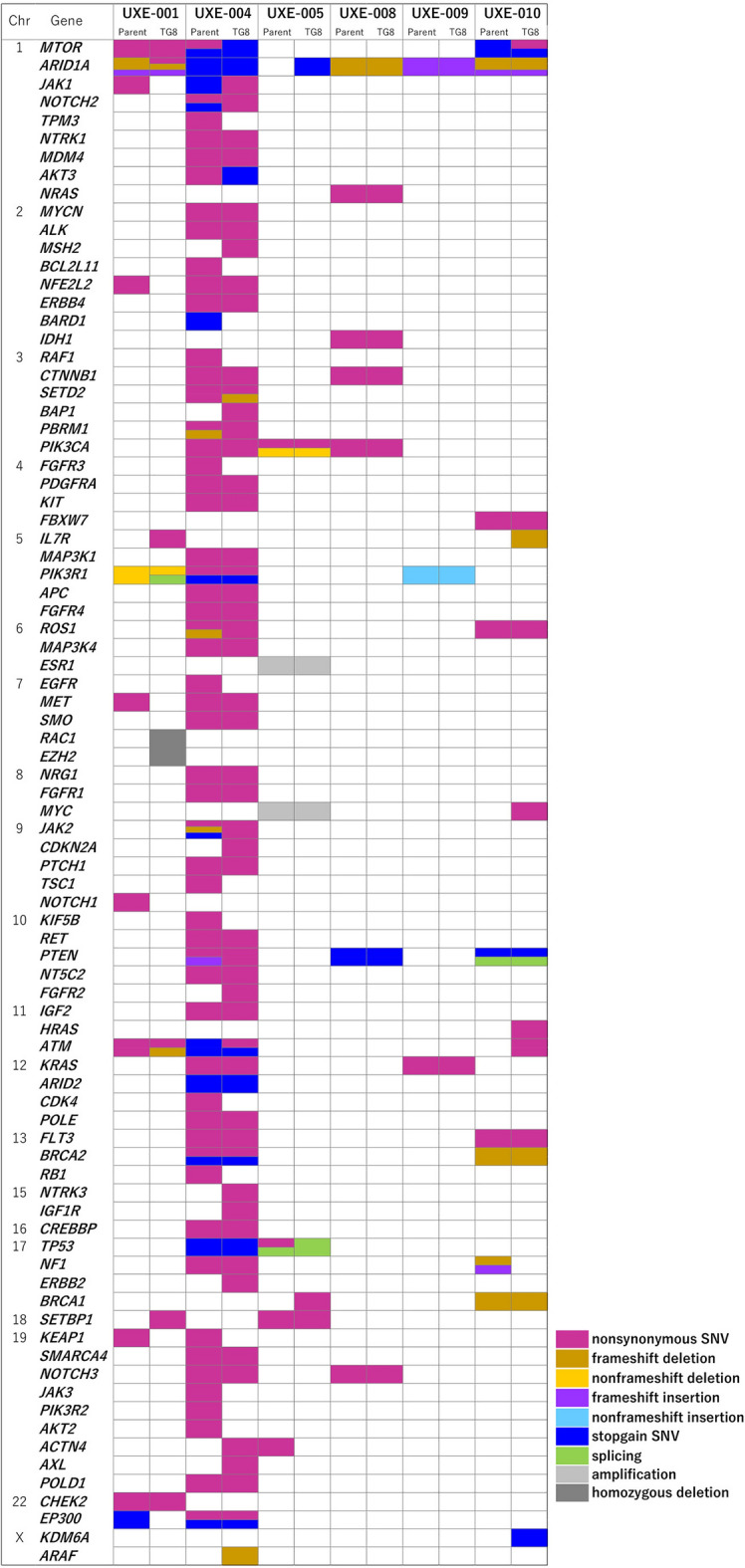
Comparison chart of gene mutations in parental endometrial cancer tissues and PDXs of six cases that reached passage TG8. Mutations in 114 cancer-related genes and amplifications and fusions in 12 genes were analyzed using the NCC Oncopanel test. Mutations at varying frequencies were observed in all six cases, a few amplifications and homozygous deletions were observed in each of the six cases, and no fusion was detected in any cases. *Parent* parental tumor tissues, *TG8* TransGeneration 8, *SNV* single nucleotide variant.

Genomic alterations in parental tissues and PDXs of six cases that reached passage TG8 were analyzed by targeted sequencing using the NCC Oncopanel test. The results are illustrated in Fig. 4, and their variant rates and amino acid change status are summarized in Supplementary Table S2. Variations were found in parental tissues and TG8-PDXs in all the cases analyzed, and the number of mutated genes was highest in parent tissues and PDXs of case UXE-004, in which an exonuclease domain mutation (EDM; V411L) in the replicative DNA polymerase epsilon (*POLE*) was included, suggesting that this case could be classified as *POLE* ultramutated endometrial cancer^37,38^. Variants in several genes, including frameshift deletion/insertion in *ARID1A* observed in the parental tumor tissue, were almost maintained in TG8-PDXs of cases UXE-008 and UXE-009. On the other hand, in addition to parental genomic alterations, including stop-gain mutations in *ARID1A* and *AKT3* in TG8-PDXs of cases UXE-005 and UXE-004, respectively, frameshift deletions in *IL7R* and *ARAF* in TG8-PDXs of cases UXE-010 and UXE-004, respectively, and nonsynonymous mutations in *MYC* and *HRAS* in TG8-PDXs of case UXE-010 were found in PDXs. The single nucleotide variant in *MYC* was observed in early PDX passages, and the frameshift deletion in IL7R was found in late PDX passages for the case of UXE-010 (Supplementary Fig. S4). Infrequent variations in several genes in the parental tumor tissues were not observed in TG8-PDX, suggesting a negative selection of the variant clones.

### Possible reduction of reactivity to anticancer drugs in endometrial-carcinoma PDXs via passaging

Tumor volume was increased during the experimental period of 12 days by ~ 1.5–2.2-fold in the control groups of mice with TG3 and TG8 PDXs of cases UXE-009 and UXE-010, and growth inhibition by paclitaxel (20 mg/kg bodyweight, i.p., once every 3 days up to 5 times) was confirmed in the mice with TG3 PDX of cases UXE-009 and UXE-010 and mice with TG8 PDXs of case UXE-009 (Supplementary Fig. S5). However, growth inhibition by paclitaxel was greatly diminished among the mice with TG8 PDXs of case UXE-010 treated with paclitaxel (Supplementary Fig. S5). The effects of paclitaxel were observed histopathologically, and apoptosis and mitotic arrest occurred in tumor cells in both UXE-009 and UXE-010 (Supplementary Fig. S6).

## Discussion

To clarify the presence or absence of alterations in the molecular properties of PDXs with up to eight passages in endometrial carcinomas, surgical samples from 12 cases were implanted into female NOG or NSG mice; those from 6 cases were successfully engrafted, and PDX tissue samples were obtained. In previous studies, the success rates of endometrial cancer PDX engraftment were reported to be 57–60%^19,20^, which were consistent with the present results. We conducted the first comparative assessment of histopathological characteristics between parental tumors and PDX samples from early and late generations. As one of the most striking findings on the morphological analysis, PDXs from carcinosarcomas, which consisted of both carcinoma and sarcomatous components in parental tumors, predominantly consisted of sarcomatous components in PDXs of TG1 or later generations in a total of 11 (73%) mice, regardless of distribution of components in the parental cases. In contrast, PDXs from endometrioid carcinomas maintained their morphological characteristics throughout the experiment, i.e., from TG1 to TG8. This result is partly consistent with a previous report^20^. Formerly, it was thought that two independent tumors (a carcinoma and a sarcoma) collided (collision theory) to for carcinosarcomas; alternatively, a combination of cellular masses could have undergone early divergence from a common precursor stem cell (combination theory). In the late 1990s, several immunohistochemical and/or genomic studies demonstrated that most carcinosarcomas were monoclonal in origin, with late divergence of the carcinoma into the sarcomatous component (conversion theory)^39,40^, and more recently, molecular integrated analyses demonstrated that carcinosarcomas exhibited phenotypic diversity in terms of epithelial-mesenchymal transition (EMT) that distinguishes them from other gynecologic cancers, such as endometrioid endometrial carcinomas and ovarian serous carcinomas^41^. The cause of the predominance of the sarcomatous component in PDXs compared to their parental tumors is not clear; however, there is a possibility that a positive selection of aggressive sarcomatous components resulted in predominant growth of those components in PDXs.

When considering therapeutic target molecules in PDX, the predominance of a sarcomatous component in carcinosarcomas is expected to be a cause for concern. This is because therapeutic target molecules that tend to be positive for the carcinoma component (e.g., HER2 and other molecules expressed in the epithelium^42^) may be underestimated in PDXs with a predominant sarcomatous component. This could result in the premature termination of therapeutic development, such as in the drug efficacy evaluation of antibody-based drugs. Carcinosarcoma is relatively infrequent but has a poor prognosis and has attracted much attention in therapeutic development. Thus, there are high expectations for the use of PDXs. Our findings reveal important pitfalls to consider in the use of PDXs for uterine carcinosarcoma.

Alterations in the immunohistochemical characteristics of ER, PTEN, PAX8, and PAX2 were noted in PDXs and their passages regardless of the parental tumor phenotypes, i.e., endometrioid carcinomas and carcinosarcomas. PAX8 is used as a marker to distinguish carcinoma of gynecologic origin from carcinomas originating from other organs and mesothelioma^43–45^. PAX2 is well known to play a major role in the development of both ductal and mesenchymal components of the urogenital system^46^. PAX2 also plays a role in the development of Müllerian-derived organs, including the uterus^47^. PAX8 and PAX2 have been suggested to play endometrial protumorigenic roles^33,47^. Therefore, conversion to PAX8-positive cells in PDXs from two cases (UXE-004 and UXE-005) was speculated to be consistent with the stimulated growth of PDXs in later passages. On the other hand, the cause of conversion to PAX8- or PAX2-negativity in PDXs from the other two cases (UXE-008 for PAX8 and UXE-001 and UXE-008 for PAX2) was not clear. A low positivity rate for PAX8 in sarcomatous and undifferentiated components was demonstrated in uterine carcinosarcoma cases^48^, suggesting that the conversion to PAX8- or PAX2-negative cells might be a result of dedifferentiation or EMT of tumor cells in PDXs. The alteration in ER expression according to immunohistochemistry of PDXs from two cases (UXE-001 and UXE-008) was suggested to be partly associated with the abovementioned mechanisms, e.g., cellular dedifferentiation. The proportion of PTEN-positive cells was almost maintained in each PDX compared to the parental tumor in one case (UXE-009) and increased in another case (UXE-005). Endometrial carcinomas have been demonstrated to have more frequent mutations in the PI3K/AKT pathway than any other tumor^49^. On the other hand, mutual exclusiveness between *KRAS* and a PI3K/AKT pathway gene involving *PIK3CA* mutations was reported in endometrial carcinoma^50^. Therefore, further study is required to elucidate the mechanisms underlying the selection of PTEN-positive cells in PDXs in relation to molecular changes in other signaling pathways. Accurate classification of the parental tumors and PDXs into molecular subgroups cannot be performed, since mismatch repair protein (MMR) IHC was only conducted for a limited number of proteins, e.g., PMS2 and MSH6^51^; however, MMR IHC abnormality was noted in the parental tumor, and it was maintained in PDXs of case UXE-010. In another case, UXE-004, loss of PMS2 was found in the parental tumor, and the status was unclear due to poor staining in PDXs with later passages. An EDM mutation in *POLE* was observed in the parental tumor and TG8-PDXs of case UXE-004. When assessing TP53 status as a surrogate for copy number, the parental tumor of the case UXE-005 was indicated to have p53 copy number alteration; however, the status was observed as wild type in PDXs, which may be due to the phenotype implanted tissues in mice. Overall, some of the representative genomic/phenotypic alterations that are used as the basis for the molecular classification of endometrial cancer were affected during the establishment and passaging of PDXs.

In a targeted genomic sequence analysis of parental tumors and PDX tissues, additional genomic alterations in cancer-associated genes were observed in PDXs for four of six cases; an EDM mutation in *POLE* was observed in the parental tumor tissues of one case (UXE-004), and a frameshift deletion (K654fs*47) in *BRCA1* was found in the parental tumor tissue of another case (UXE-010). *BRCA1* is a DNA repair gene frequently mutated in low-grade endometrioid tumors^52^, and the additional genomic alteration in PDXs was considered to be associated with the mutation in *BRCA1*. For the case UXE-010, loss of MSH6 was noted in the immunohistochemical analysis; genomic alterations in MSH6 could accumulate during the passaging of PDXs^25^. Paclitaxel responsiveness was observed to varying degrees among mice with TG8 PDXs, and the results were similar for mice with TG3 PDXs of case UXE-010. These results suggested that accumulated gene mutations in PDXs with later passages were implicated in heterogeneous growth/responsiveness to the drug. Similar variation in growth rates was noted among PDXs at each transplant generation of UXE-10, consistent with the accumulation of gene mutations in the PDXs. Overall, immunohistochemical alterations and additional genomic alterations were not clearly associated with each other in PDXs for the cases UXE-001, UXE-004, UXE-005, and UXE-010. Therefore, analysis of both protein and genomic markers should be conducted to clarify differences in the characteristics between PDXs and parental tumors. In previous reports, the genomic profiles of PDXs of uterine carcinomas were demonstrated to be stable compared to those of parental tumors^19,20^; however, the extent of alterations in the molecular properties between endometrial cancers and PDX models varied in the present study.

One of the limitations of the present study is that a minimal number of cases were analyzed, particularly for drug-response changes. One analyzed case was a typical tumor with carcinosarcomatous phenotype, and the other case had an endometrioid phenotype. In addition, the most frequent single nuclear variations in *TP53* in endometrial carcinomas^41,49^ were observed in the parental tissues of only two cases analyzed. Therefore, further study is needed to elucidate these unresolved issues.

In summary, three novel findings were observed in the present experiments using PDXs from endometrial cancer cases:

1. In carcinosarcoma cases, sarcomatous components in the parental tumors became predominant in PDXs. This was suggested to be a result of positive selection of aggressive sarcomatous components.
2. Conversion to PAX8-negative cells in PDXs of one carcinosarcoma case or to PAX2-negative cells in PDXs of two endometrioid carcinoma and one carcinosarcoma cases might be a result of dedifferentiation or epithelial–mesenchymal transition of tumor cells.
3. Mutations in chromatin remodeling and/or DNA repair genes such as *BRCA1* and *POLE*, which are frequently observed in endometrioid tumors, are considered to accumulate additional gene mutations in later passages. Such genomic alterations may be implicated in the heterogeneous responsiveness to the drug.

Overall, the genomic and phenotypic alterations observed between endometrial carcinoma PDXs and parental tumors were partly associated with endometrial cancer-specific characteristics related to cellular differentiation and gene mutations.

## Methods

### Tissue sampling

A total of 12 surgical specimens of endometrial carcinomas were obtained between August 2017 and December 2017 at the National Cancer Center Hospital (Table 1). The clinical data and samples were handled in accordance with relevant domestic guidelines and regulations in Japan. The use of patients’ surgical specimens in this study was approved by the ethics committee of the National Cancer Center, Tokyo, Japan (2015-108), and written informed consent was obtained from all patients. After sampling for pathological evaluation, the samples were stored on ice, and each sample was dissected into approximately 2–3-mm cubes and used for the establishment of PDXs (numbered from UXE-001 to UXE-012). The remaining fragments were simultaneously frozen in liquid nitrogen and stored at − 80 °C for the isolation of DNA or fixed with 10% buffered formalin for the preparation of tissue sections for morphological identification of PDX-originated tumor tissues.

### Establishment of PDX

Surgical specimens from the 12 cases cut into approximately 2–3 mm cubes were implanted into the subcutis of 4 female NOD. Cg*Prkdc*^*scid*^*Il2rg*^*tm1Sug*^/ShiJic (NOG) mice (In-Vivo Science, Kawasaki, Japan) or NOD. Cg-*Prkdc*^*scid*^*Il2rg*^*tm1WjI*^/SzJ (NSG) mice (The Jackson Laboratory Japan, Yokohama, Japan) per case under isoflurane anesthesia (Zoetis Japan, Tokyo, Japan). The mice were housed in plastic cages with recycled paper bedding (n ≤ 5 per cage) in a specific pathogen-free environment maintained at 22 ± 1 °C and 55 ± 10% relative humidity in a 12:12-h light/dark cycle and given free access to the standard chow diet CE2 (CLEA Japan, Tokyo, Japan) and tap water. Mouse experiments were carried out at the Animal Facility of the National Cancer Center Research Institute (Tokyo, Japan) according to domestic and institutional guidelines and the ARRIVE guidelines 2.0 and following the approval of the National Cancer Center Animal Ethics Committee (approval no. T17-006). The implanted region of each mouse was palpated weekly, and the size of subcutaneous nodules was measured using calipers. A successfully engrafted tumor was designated Transplant Generation 1 (TG1). When the longest nodule diameter measured using calipers exceeded 10 mm, the mice were euthanized by isoflurane overdose, and the subcutaneous nodules were excised, followed by transplantation of specimens cut into approximately 2–3 mm cubes into other NOG mice; part of the specimens cryopreserved in LaboBanker 2 medium (TOSC Japan, Tokyo, Japan) and stored at − 80 °C to save PDX stocks, and the rest were; fixed with 10% neutrally buffered formalin for preparation of tissue sections for histopathological and immunohistochemical analysis. Several pieces of the tissue were also cryopreserved for targeted genomic sequence analysis. For successfully engrafted PDXs, the passages to NOG mice of the next generation were repeated up to 7 times (up to TG8). For mice without detectable engraftment, weekly observation by palpation was continued for up to 52 weeks, followed by euthanization.

### Histopathology and immunohistochemistry

All parental tumor tissues and PDX tissues fixed in 10% neutral buffered formalin were processed routinely and embedded in paraffin wax. The sections (3 μm thick) were stained with hematoxylin and eosin (HE) and histopathologically evaluated. Paraffin-embedded sections were also used for immunohistochemistry analysis, and primary antibodies against human pancytokeratin (prediluted; clone AE1/AE3, Dako, Glostrup, Denmark), PAX8 (1:50; clone PAX8R1, Abcam, Cambridge, MA, USA), estrogen receptor (ER) (prediluted; clone SP1, Ventana Medical Systems, Tucson, AZ, USA), TP53 (prediluted; clone DO7, Dako), ARID1A (1:2000; rabbit polyclonal, Sigma, St. Louis, MO, USA), PAX2 (1:200 dilution; clone EPR8586, Abcam), PTEN (1:200; clone D4.3, Cell Signaling Technology, Beverly, MA, USA), PMS2 (prediluted; clone EP51, Dako), and MSH6 (prediluted; clone EP49, Dako) were used for all cases. Primary antibodies against human CD45 (prediluted; clone M0701, Dako) were optionally used for several cases.

### Targeted genomic sequence analysis

Genomic DNA extracted from parental tumor tissues and PDX tissues of six cases that were passaged to TG8 was prepared using the NucleoSpin Tissue kit (Takara Bio, Kusatsu, Japan) according to the manufacturer’s protocol. Using the DNA samples from parental tumors and TG8-PDXs of the six cases, a targeted sequencing analysis was performed using the NCC Oncopanel v4 test, which can analyze mutations of 114 genes and amplifications and fusions of 12 genes^53^. Procedures for targeted sequencing and data analysis were previously described^53^. We also performed Sanger sequencing analyses of DNA samples from the TG1-7- PDXs of cases UXE-009 and UXE-010. The PCR products, including mutations and deletions, were amplified using specific PCR primers, and the amplified PCR products were directly sequenced using the forward primers used in the PCR for each target. The primers used for *MYC*, 176 C>T; and *IL7R*, 793 del A are described in Supplementary Table S3.

### Drug administration and efficacy evaluation in PDXs

Engraftment of frozen PDXs from all cases was confirmed after subcutaneous reimplantation into female NOG mice. In the drug efficacy evaluation tests, frozen TG3 and TG8 samples of UXE-009 (from mouse No. 1) and UXE-010 (from mouse No. 2) were used. Ten or eight PDX mice in TG3 and TG8 (for both case UXE-009 and case UXE-010) were divided into two groups based on the bodyweight and PDX tumor volumes from 48.7 mm^3^ to 152.4 mm^3^ at the start of treatment with the microtubule inhibitor paclitaxel (Nipro, Osaka, Japan). The tumor size was measured using a caliper, and the volume was calculated as follows: Volume = (length) × (width) x (height) × π/6. Paclitaxel dissolved in saline at 6 mg/mL was intraperitoneally administered at 0 (control) and 20 mg/kg body weight after measurement of body weight and size of subcutaneous nodules once every 3 days up to 5 times. Mice were euthanized under isoflurane anesthesia 24 h after the last administration, and of the nodules were extracted for histopathology as mentioned above. The experimental endpoint was defined by excessive tumor growth (> 10% of bodyweight) or general impairment (without transient staggering after paclitaxel administration).

### Statistical analysis

The size of the subcutaneous nodules is presented as the mean ± SD. Differences between groups were analyzed by Student’s t test following the F test using EZR^54^. *P* values of < 0.05 and < 0.01 were considered significant.

## Supplementary Information

Supplementary Information.

## Data Availability

The datasets used and/or analyzed during the current study are deposited at the Japanese Genotype–phenotype Archive (JGA; https://www.ddbj.nig.ac.jp/jga), which is hosted by the Bioinformation and DDBJ Center, under accession number JGAS000585/JGAD000712.
