# Supplementary material for: Alteration in molecular properties during establishment and passaging of endometrial carcinoma patient-derived xenografts
Source: Sci Rep. 2023 May 25;13:8511. doi: 10.1038/s41598-023-35703-6 (PMC10212914; doi:10.1038/s41598-023-35703-6)
Supplement: Supplementary file 1 — Supplementary Information. [file 41598_2023_35703_MOESM1_ESM.pdf]

Case No.	Antigen	Parental tissue	TG1	TG2 – TG6	TG8
UXE-001	AE1/AE3	+	+	+	+
	ER	+	+/− or ++	−	−
	TP53	WT	WT	WT	WT
	PTEN	−	−	−	−
	ARID1A	−	−	−	−
	PAX8	+	+	+	+
	PAX2	+focal	− or +focal	−	−
	PMS2	+	NA	NA	NA
	MSH6	+	+	+	+
UXE-004	AE1/AE3	+	+	+	+/−
	ER	++	+/− or ++	++	++
	TP53	WT	WT	WT	WT
	PTEN	−	−	−	−
	ARID1A	−	−	−	−
	PAX8	+/−	+/−	+	+
	PAX2	−	−	−	−
	PMS2	−partly	− or +/−	NA	NA
	MSH6	+	+	+	+
UXE-005	AE1/AE3	+	+/−	+	+
	ER	++	+/−	++	++
	TP53	+partly	WT	WT	WT
	PTEN	−	−	+	+
	ARID1A	+	+	+	+
	PAX8	+/−	−	+	+
	PAX2	−	−	−	−
	PMS2	+	+	+	+
	MSH6	+	+	+	+
UXE-008	AE1/AE3	+/−	− or +/−	−	+/−
	ER	+/−	− or +	−	−
	TP53	WT	WT	WT	WT
	PTEN	−	−	−	−
	ARID1A	−	−	−	−
	PAX8	+	− or +	−	−
	PAX2	+focal	− or +focal	−	−
	PMS2	+	+	+	+
	MSH6	+	+	+	+
UEX-009	AE1/AE3	+/−	+/−	+/−	+/−
	ER	−	−	−	−
	TP53	WT	WT	WT	WT
	PTEN	+focal	+	+	+
	ARID1A	−	−	−	−
	PAX8	+/−	−	+/−	+/−
	PAX2	−	−	−	−
	PMS2	+	+	+	+
	MSH6	+	+	+	+
UXE-010	AE1/AE3	+	+	+	+
	ER	+	+/++	++	++
	TP53	WT	WT	WT	WT
	PTEN	−	−	−	−
	ARID1A	−	−	−	−
	PAX8	+	+	+	+
	PAX2	+focal	+focal	− or +focal	− or +focal
	PMS2	+	+	+	+
	MSH6	−	−	−	−

Supplementary Table S1 Summary of immunohistochemistry for parental tissues and PDXs

AE1/AE3, PAX8: +, positive; +/−, including negative cell groups; −, almost all negative cells

ER: ++, >50%; +, 10-50%; +/−, 1-10%, −, 0-1% positive cells

TP53: WT, scattered positice cells ; +, mutant patterns with almost all positive cells

ARID1A, MSH6: +, retained; −, lost

PAX2, PTEN: +, focal, including negative cell groups; −, almost all negative cells

PMS2: +, retained; +/−, including negative cell groups; −, lost; NA, poor staining

Case No.	Gene Name	Variant Function	AA change	Variant rate (%)	
				Parental tissue	PDX-TG8
001	*MTOR*	nonsynonymous SNV	G305C	4.3	
			K1815T		10.4
	ARID1A	frameshift deletion	S634fs*12	9.5	40.8
		frameshift insertion	Y1027*	14.7	47.7
		nonsynonymous SNV	R1833C		22.4
	JAK1	nonsynonymous SNV	R542C	4.4	
	NFE2L2	nonsynonymous SNV	E82D	4.2	
	IL7R	nonsynonymous SNV	N65S		27.6
	PIK3R1	nonframeshift deletion	I405del	7.5	39.6
		splicing	—		39.7
	MET	nonsynonymous SNV	M1229V	3.4	
	NOTCH1	nonsynonymous SNV	E681D	3.6	
	ATM	nonsynonymous SNV	R2832H	8.8	50.8
		nonframeshift deletion	F627fs*22		26.7
	SETBP1	nonsynonymous SNV	R1456C		38.1
	KEAP1	nonsynonymous SNV	R354W	1.7	
	CHEK2	nonsynonymous SNV	R474H	4.0	
			R145Q	12.2	51.9
	EP300	stopgain SNV	R202*	3.9	

Case No.	Gene Name	Variant Function	AA change	Variant rate (%)	
				Parental tissue	PDX-TG8
004	*MTOR*	nonsynonymous SNV	A2532V	41.5	
		stopgain SNV	G845*	2.9	50.1
	ARID1A	stopgain SNV	R1989*	89.9	99.9
	JAK1	nonsynonymous SNV	R873H	2.6	47.7
			R93H		50.1
	NOTCH2	stopgain SNV	G929*	42.2	
		nonsynonymous SNV	C547R	45.6	49.5
	TPM3	nonsynonymous SNV	A203V	33.8	
	NTRK1	nonsynonymous SNV	E413K	49.2	49.9
	MDM4	nonsynonymous SNV	A472T	2.4	48.4
	AKT3	nonsynonymous SNV	P69Q	33.3	
		stopgain SNV	Y312*		48.2
	MYCN	nonsynonymous SNV	P44L	55.7	61.8
	ALK	nonsynonymous SNV	S1561L	42.0	44.8
			P1551S	2.5	57.0
	MSH2	nonsynonymous SNV	G204E		47.0
	BCL2L11	nonsynonymous SNV	G156R	43.0	
	NFE2L2	nonsynonymous SNV	F70C	44.2	50.1
			V591G		48.6
	ERBB4	nonsynonymous SNV	E1121D	43.6	49.6
			T731M		49.5

004	*BRAD1*	stopgain SNV	R641*	1.8	
	RAF1	nonsynonymous SNV	K439E	41.6	
	CTNNB1	nonsynonymous SNV	R225H	43.3	
			A522T	2.7	48.1
			M735L	46.6	49.9
			K133N		49.8
	SETD2	nonsynonymous SNV	P2379L	44.8	50.7
			D790Y	3.0	48.7
		frameshift insertion	R1407fs*8		37.0
	BAP1	nonsynonymous SNV	S341N		46.5
	PBRM1	frameshift deletion	F1018fs*15	36.4	
		nonsynonymous SNV	D901G	45.5	48.9
	PIK3CA	nonsynonymous SNV	E81K	51.4	55.6
	FGFR3	nonsynonymous SNV	G493S	40.5	
	PDGFRA	nonsynonymous SNV	G898D	2.0	48.7
	KIT	nonsynonymous SNV	D419N	44.2	49.1
	MAP3K1	nonsynonymous SNV	S1299R	47.6	50.4
	PIK3R1	stopgain SNV	R386*	46.3	51.7
		nonsynonymous SNV	D560G	44.5	49.3
	APC	nonsynonymous SNV	A501D	1.8	48.7
			P1665H		47.6
	FGFR4	nonsynonymous SNV	C271Y	1.4	44.1
	ROS1	nonsynonymous SNV	R2210I	46.4	47.3
		frameshift deletion	N1767fs*22	36.5	
	MAP3K4	nonsynonymous SNV	V562A	40.9	
			G857E		47.4
	FGFR	nonsynonymous SNV	R671C	35.4	
	MET	nonsynonymous SNV	S752Y	47.3	48.8
			V1362I	42.0	
			P210T		49.0
	SMO	nonsynonymous SNV	T336A	49.7	60.4
	NRG1	nonsynonymous SNV	A531A	41.8	
			R220H		50.1
	FGFR1	nonsynonymous SNV	R250W	1.8	46.7
	JAK2	frameshift deletion	K302fs*8	35.8	
		nonsynonymous SNV	V392M	2.6	50.6
		stopgain SNV	E1006*	42.1	
	CDKN2A	nonsynonymous SNV	G67D		36.1
	PTCH1	nonsynonymous SNV	S1359R	33.5	
			L254S		47.8
	TSC1	nonsynonymous SNV	V219A	36.6	
	KIF5B	nonsynonymous SNV	Y649H	42.0	
	RET	nonsynonymous SNV	F195L	2.5	49.0
	PTEN	nonsynonymous SNV	R130Q	46.2	47.9
		frameshift insertion	N323fs*2	41.3	
	NT5C2	nonsynonymous SNV	R367Q	44.7	52.6
	FGFR2	nonsynonymous SNV	A53V		43.1

004	*IGF2*	nonsynonymous SNV	G49S	80.8	99.7
	ATM	stopgain SNV	R1730*	47.1	48.6
		nonsynonymous SNV	S978Y		51.5
	KRAS	nonsynonymous SNV	G12A	42.9	65.1
	ARID2	stopgain SNV	Q965*	2.0	48.4
	CDK4	nonsynonymous SNV	K106N	41.9	
	POLE	nonsynonymous SNV	S1931F	2.6	47.1
			V411L	34.5	39.7
	FLT3	nonsynonymous SNV	Y597C	3.0	49.4
			F316V	2.5	47.2
	BRCA2	stopgain SNV	E790*	45.3	48.6
		nonsynonymous SNV	I1065S	46.6	51.2
	RB1	nonsynonymous SNV	R857C	42.8	
	NTRK3	nonsynonymous SNV	R536K		49.9
	IGF1R	nonsynonymous SNV	V732M		46.0
	CREBBP	nonsynonymous SNV	R1786C	2.0	44.9
			R624C	2.0	48.7
	TP53	stopgain SNV	R196*	45.2	47.6
	NF1	nonsynonymous SNV	V212F	2.2	49.7
	ERBB2	nonsynonymous SNV	R968I		49.0
	KEAP1	nonsynonymous SNV	D165N	43.2	
	SMARCA4	nonsynonymous SNV	E861K	45.4	49.9
	NOTCH3	nonsynonymous SNV	V905M	2.0	47.7
			A564T	38.0	41.4
	JAK3	nonsynonymous SNV	E1019D	33.1	
	PIK3R2	nonsynonymous SNV	A190V	62.2	
	AKT2	nonsynonymous SNV	Q472R	45.2	
	ACTN4	nonsynonymous SNV	R95W		44.8
	AXL	nonsynonymous SNV	A848V		46.5
	POLD1	nonsynonymous SNV	A308V	2.2	53.1
			T747A	3.3	52.7
	EP300	nonsynonymous SNV	P428S	44.1	49.5
		stopgain SNV	R1312*	2.4	43.2
	ARAF	frameshift deletion	R255fs*37		40.5

Case No.	Gene Name	Variant Function	AA change	Variant rate (%)	
				Parental tissue	PDX-TG8
005	*ARID1A*	stopgain SNV	Y1027*		48.4
	PIK3CA	nonsynonymous SNV	C378Y	61.5	67.0
		nonframeshift deletion	E453del	53.0	58.6
	TP53	nonsynonymous SNV	G245S	40.9	
		splicing	—	43.4	99.9
	BRCA1	nonsynonymous SNV	P1136S		46.0
	SETBP1	nonsynonymous SNV	S1492R	45.9	47.8
	ACTN4	nonsynonymous SNV	R695L	43.7	

Case No.	Gene Name	Variant Function	AA change	Variant rate (%)	
				Parental tissue	PDX-TG8
008	*ARID1A*	frameshift deletion	D1850fs*33	81.4	90.3
	NRAS	nonsynonymous SNV	Q61L	47.9	49.1
			G12D	46.3	49.8
	IDH1	nonsynonymous SNV	R132H	62.8	64.3
	CTNNB1	nonsynonymous SNV	Y333F	49.4	50.3
	PIK3CA	nonsynonymous SNV	R88Q	57.0	58.0
			E726K	46.3	48.6
	PTEN	stopgain SNV	R130*	31.2	29.0
		splicing	—	63.7	65.0
	NOTCH3	nonsynonymous SNV	N1597K	51.5	49.7

Case No.	Gene Name	Variant Function	AA change	Variant rate (%)	
				Parental tissue	PDX-TG8
009	*ARID1A*	frameshift insertion	H203fs*197	41.2	43.1
		nonframeshift deletion	P21delP		31.8
	PIK3R1	nonframeshift insertion	N453_T454insN	25.5	32.0
	KRAS	nonsynonymous SNV	G12V	34.5	47.4

Case No.	Gene Name	Variant Function	AA change	Variant rate (%)	
				Parental tissue	PDX-TG8
010	*MTOR*	stopgain SNV	R2018*	35.8	50.2
		nonsynonymous SNV	R2060W		16.1
	ARID1A	franeshift deletin	D1850fs*33	31.9	38.8
		frameshift insertion	D1850fs*4	40.8	58.0
	FBXW7	nonsynonymous SNV	R689W	35.4	49.2
	IL7R	frameshift deletion	R267fs*28		43.4
	ROS1	nonsynonymous SNV	G1915R	37.5	51.9
	MYC	nonsynonymous SNV	A59V		44.0
	PTEN	stopgain SNV	R233*	38.6	48.2
	HRAS	nonsynonymous SNV	A59T		46.5
	ATM	nonsynonymous SNV	R1973I		47.7
	FLT3	nonsynonymous SNV	T432M	49.4	49.9
	BRCA2	frameshift deletion	S1650fs*20	34.6	45.7
	NF1	frameshift deletion	Y628fs*3	14.9	
		frameshift insertion	I679fs*21	22.9	
	BRCA1	frameshift deletion	K654fs*47	35.5	42.7
	KDM6A	stopgain SNV	R393*		44.1

Supplementary Table S2 Variant rates of cancer-associated genes between original tumor tissues and TG8-PDXs.

Gene	NCBI RefSeq	Forward primer	Reverse primer
MYC	NM_002467	CCCTCAACGTTAGCTTCACC	AGCAGCTCGAATTTCTTCCA
IL7R	NM_002185	TGCATGGCTACTGAATGCTC	GCCTTAATCCCCTTTGTGGT

Supplementary Table S3 Primer sequences (5' →3') for Sanger sequencing

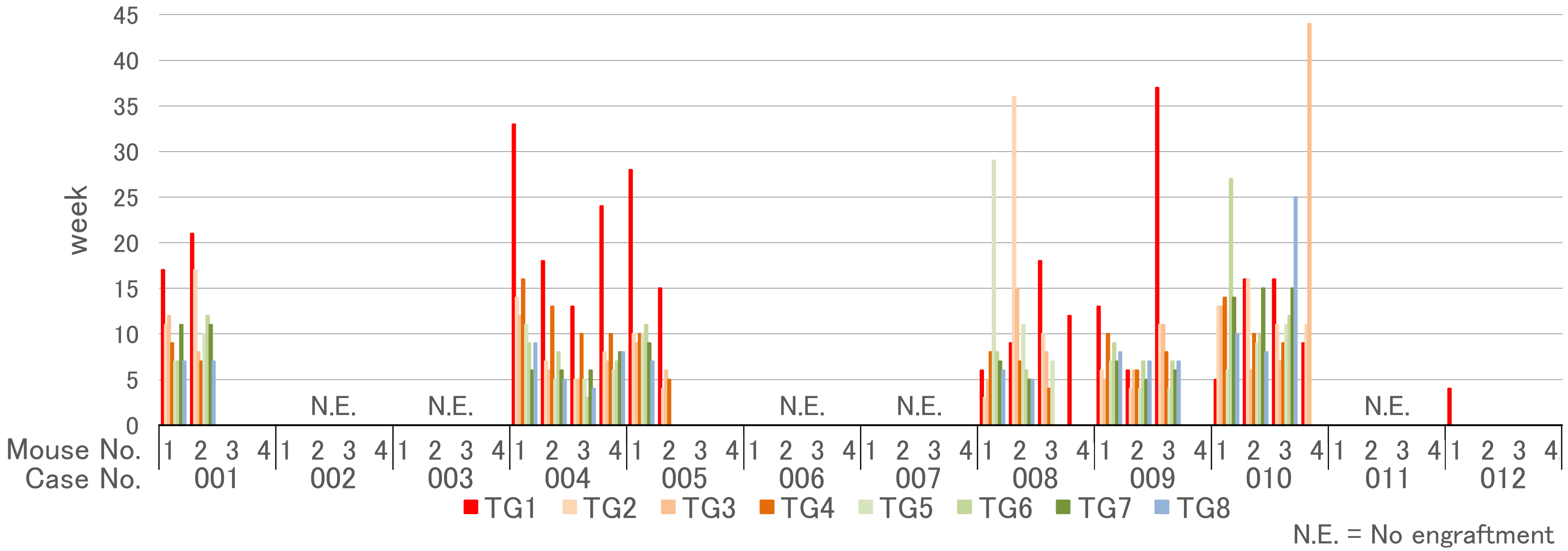

Supplementary Figure S1 Duration from implantation to passaging to the next generation, which could represent the growth of PDXs) in each mouse

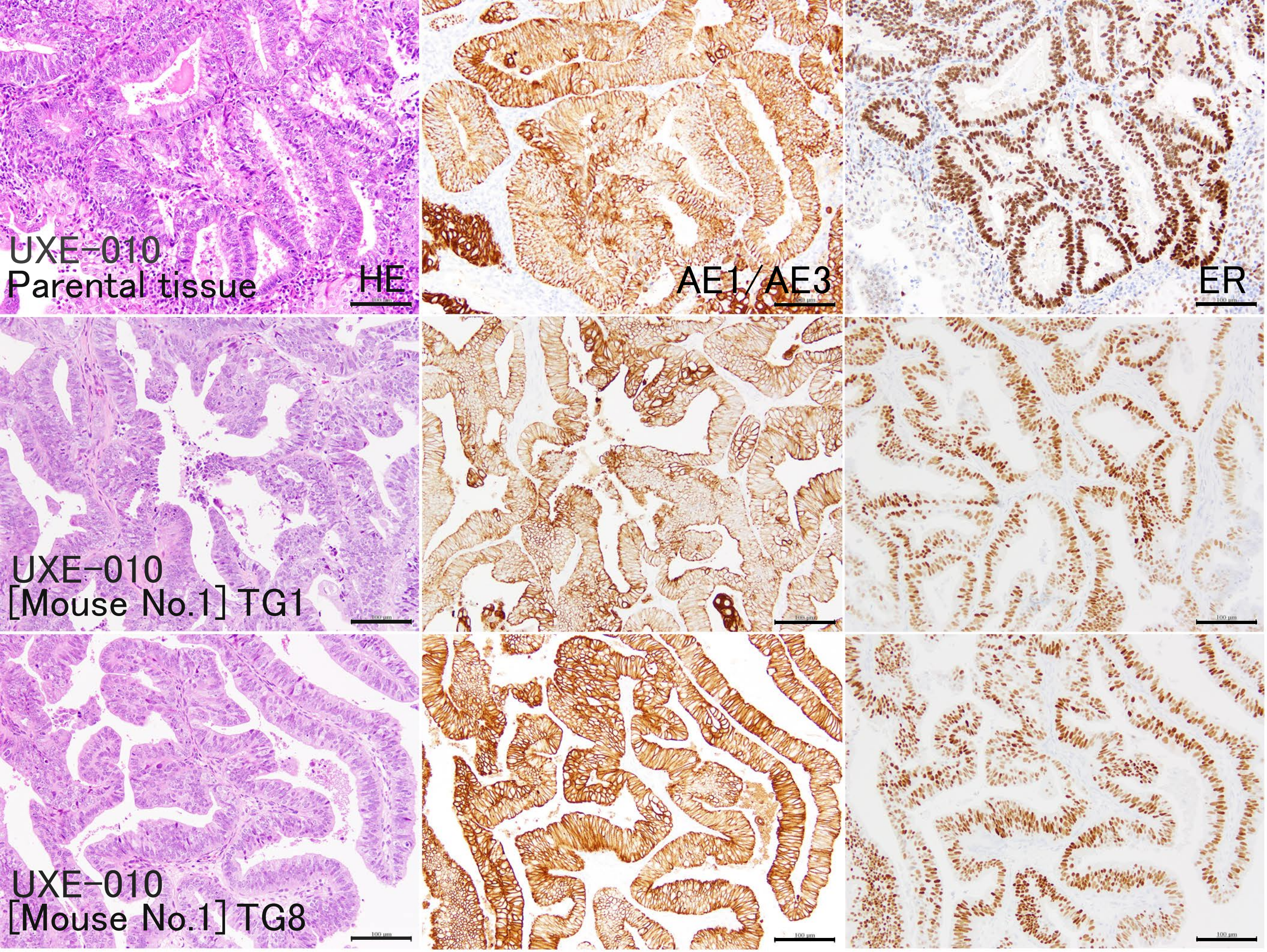

Supplementary Figure S2A

Histological and immunohistochemical photographs of a parental tissue and PDXs. Left panels, HE staining. Middle and right panels present immunohistochemical staining, using a serial section of the each left HE stained setcion.

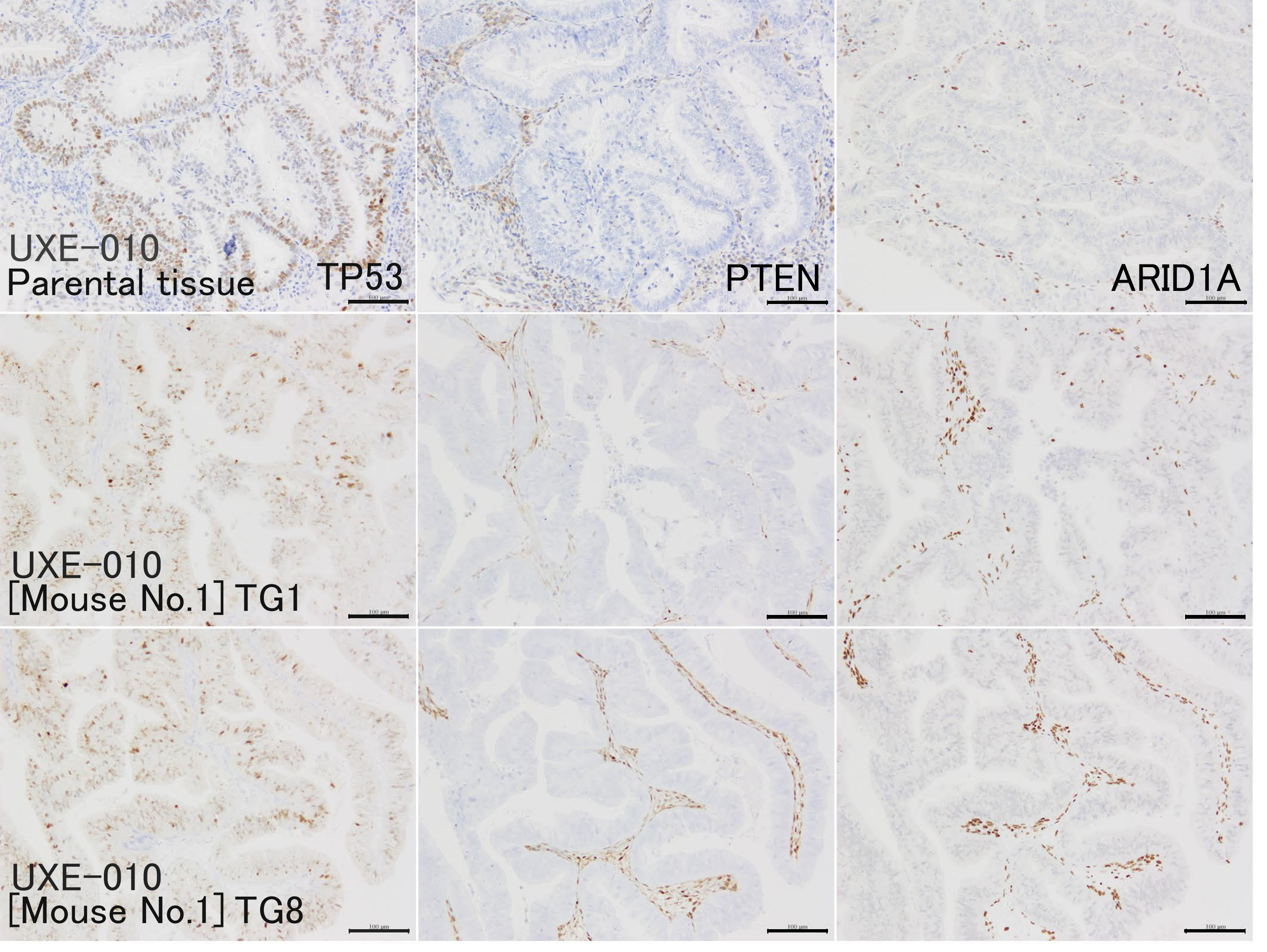

Supplementary Figure S2B

Immunohistochemical photographs of a parental tissue and PDXs, using a serial section of the each HE stained setcion.

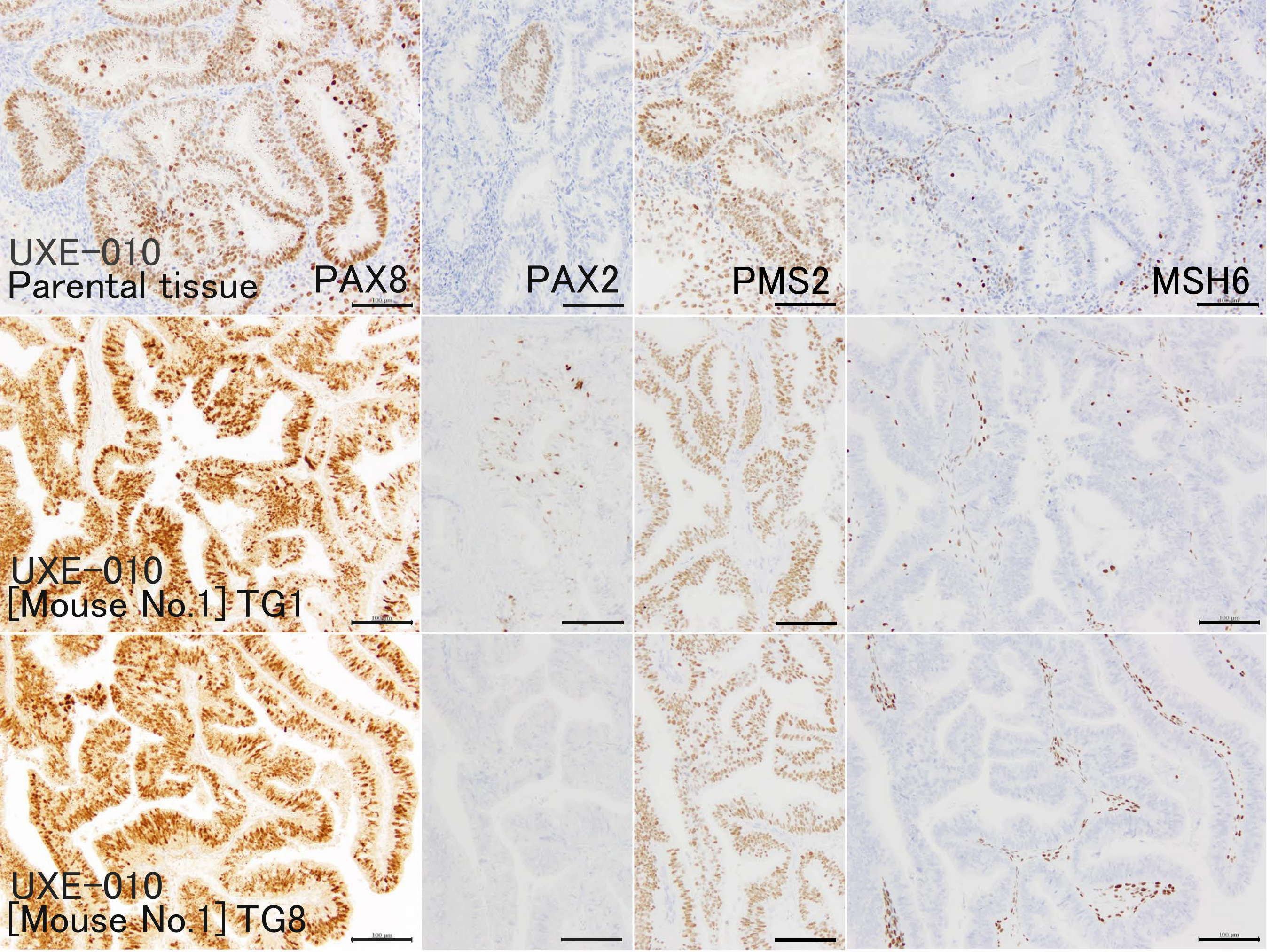

Supplementary Figure S2C

Immunohistochemical photographs of a parental tissue and PDXs, using a serial section of the each HE stained setcion.

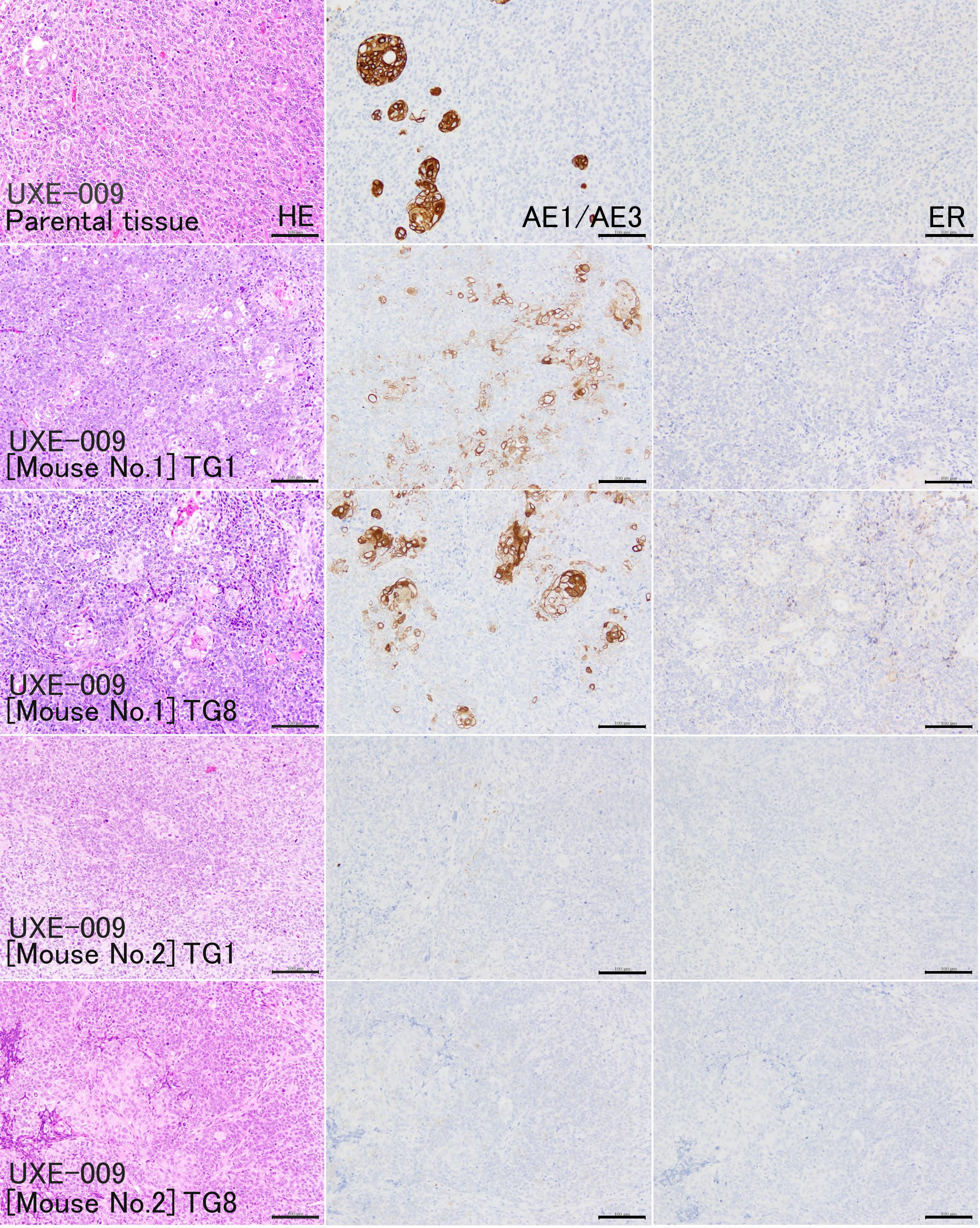

Supplementary Figure S3A

Histological and immunohistochemical photographs of a parental tissue and PDXs. Left panels, HE staining. Middle and right panels present immunohistochemical staining, using a serial section of the each left HE stained setcion.

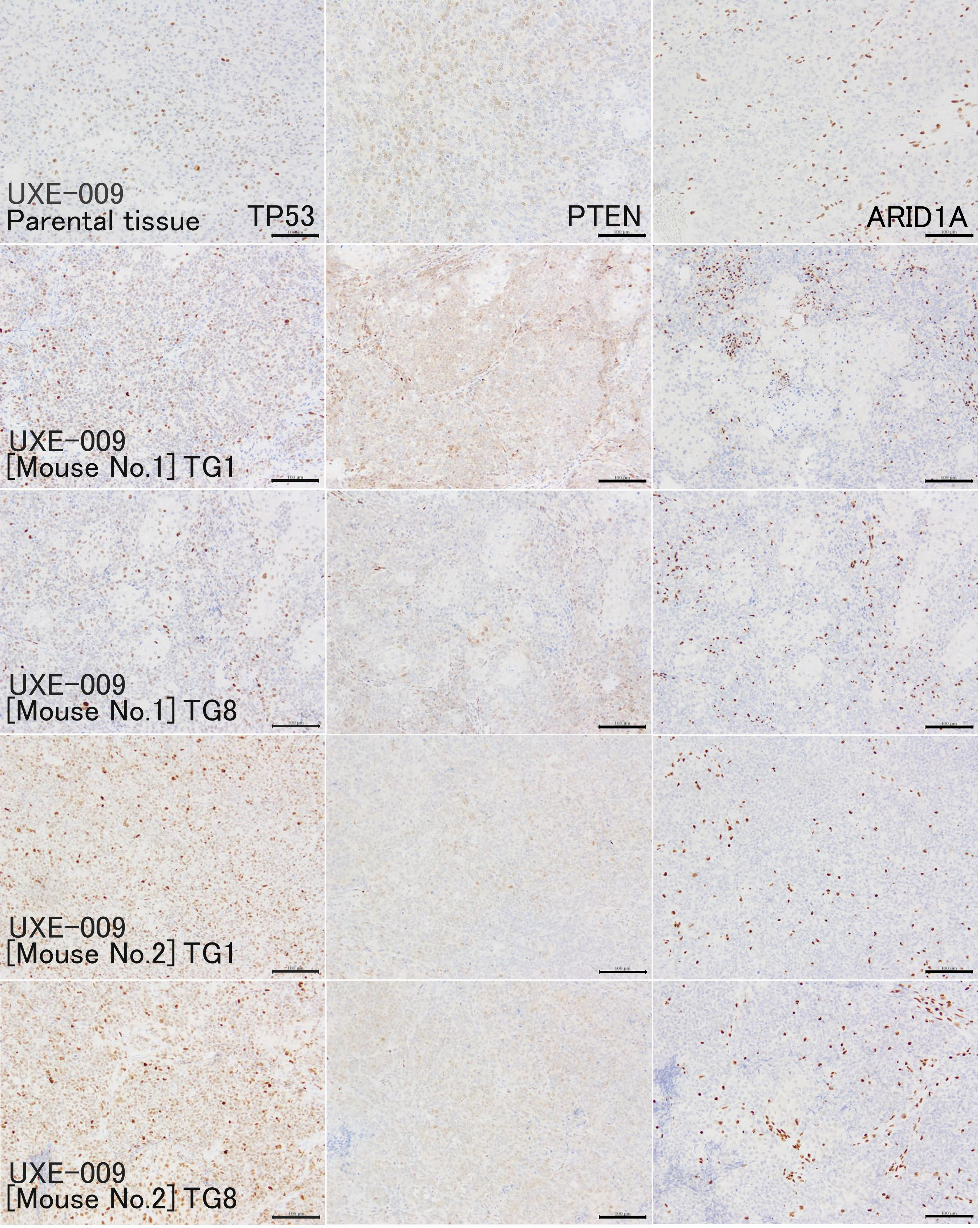

Supplementary Figure S3B

Immunohistochemical photographs of a parent tissue and PDXs, using a serial section of the each HE stained setcion.

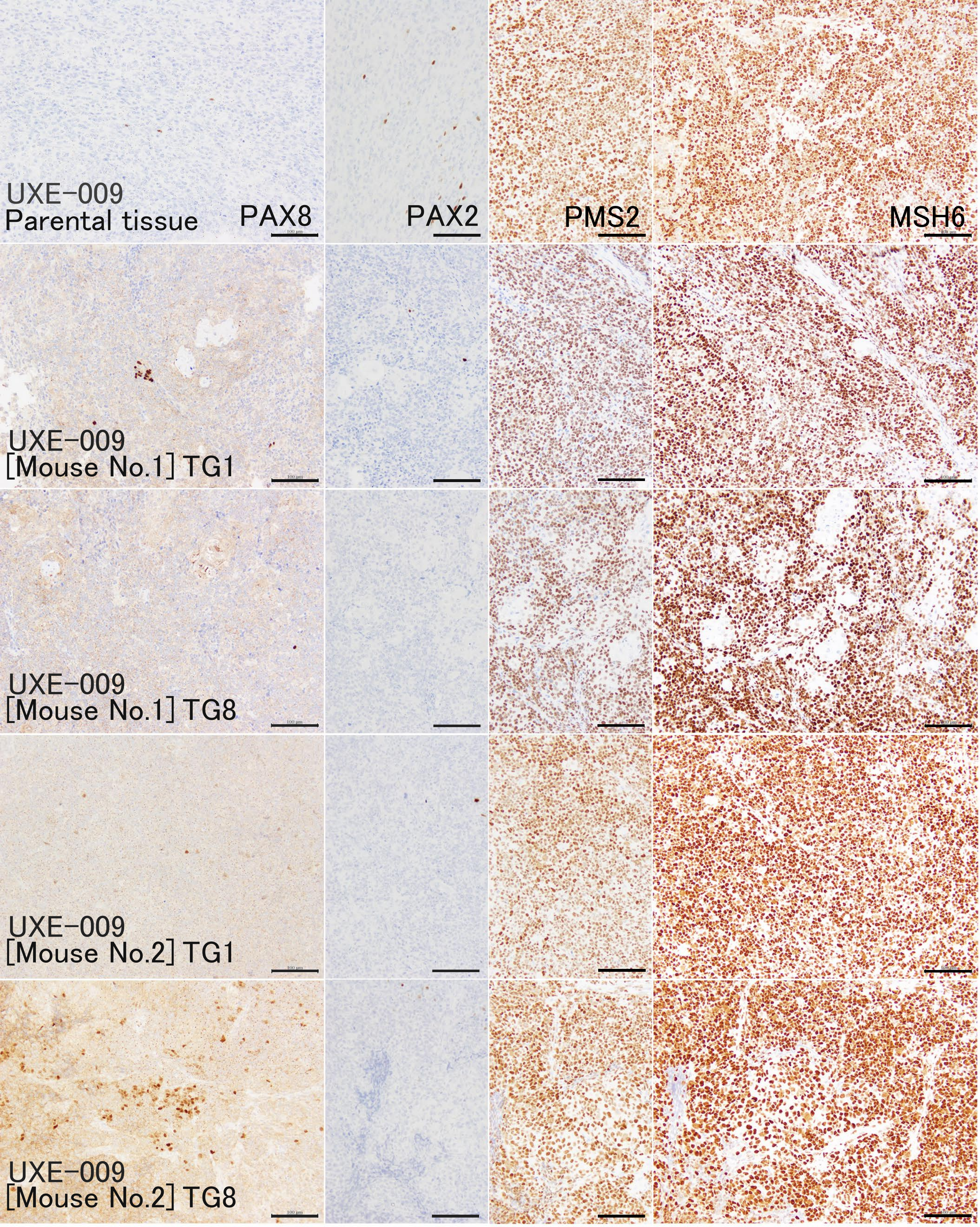

Supplementary Figure S3C

Immunohistochemical photographs of a parental tissue and PDXs, using a serial section of the each HE stained setcion.

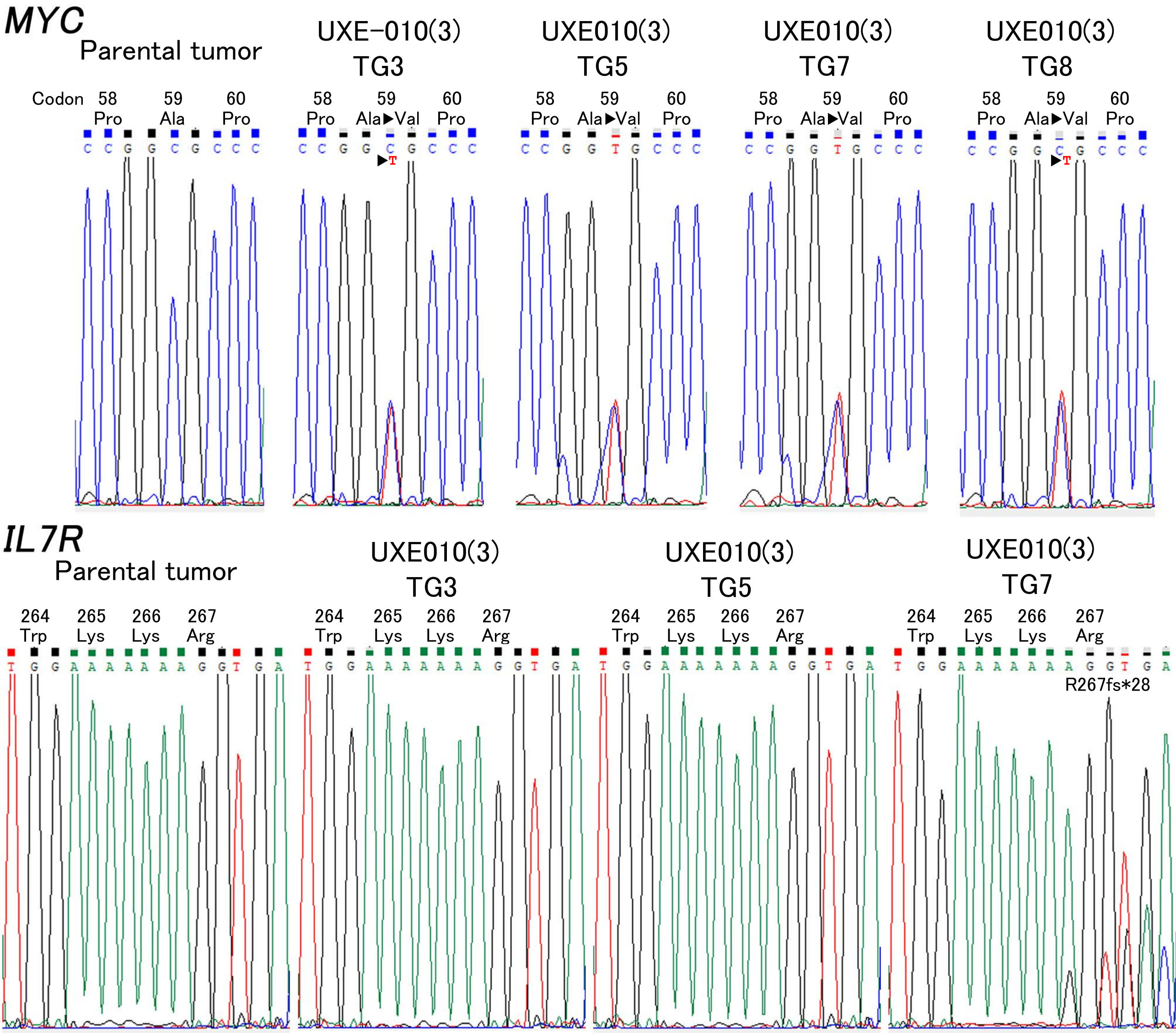

Supplementary Figure S4 Changes in variants of principal cancer-associated genes between original tumors and UXE-010-PDXs

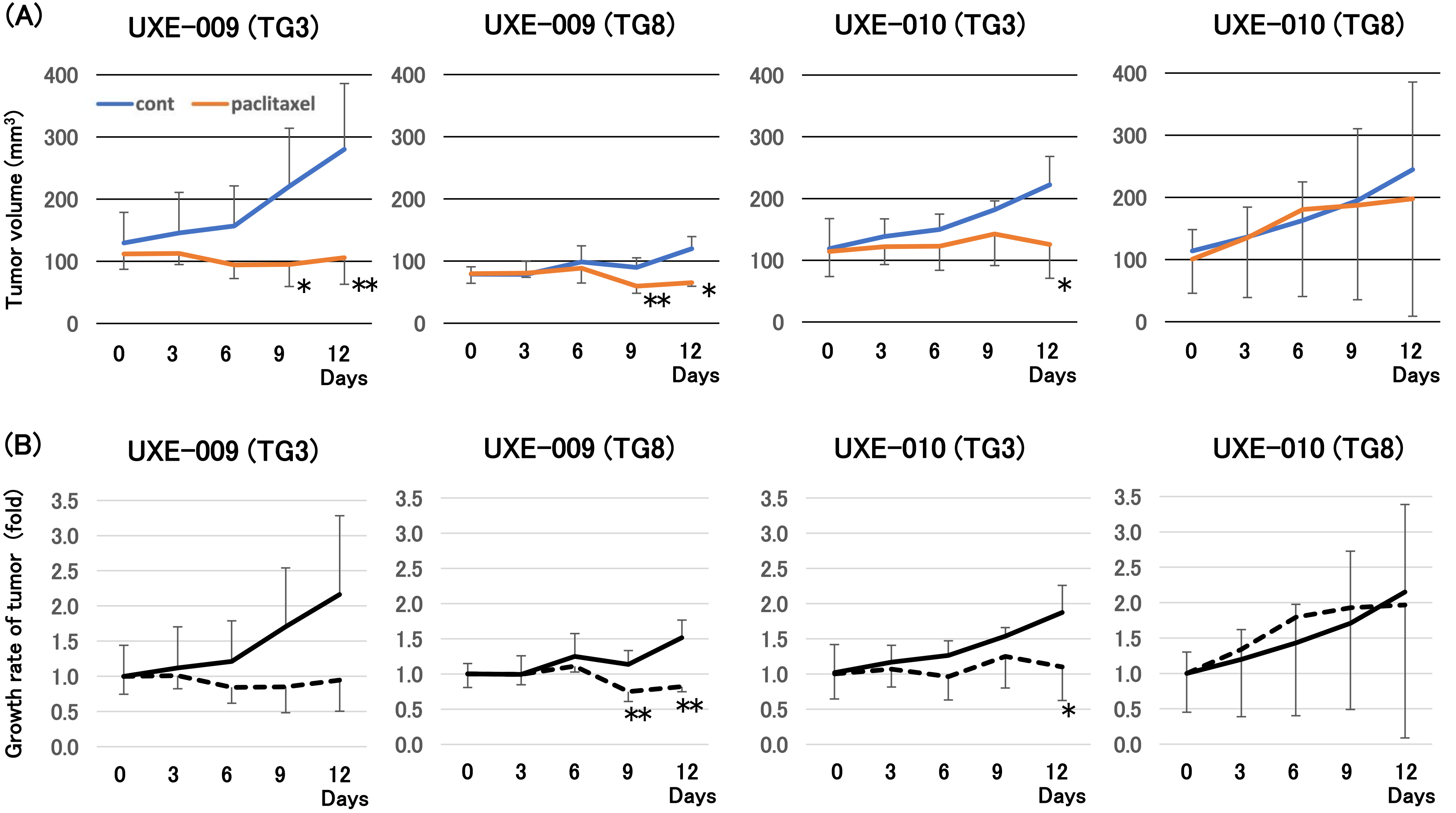

Supplementary Figure S5. Growth curves of PDXs of the control and paclitaxel (20 mg/kg bodyweight, intraperitoneally, once three days up to 5 times)-treated mice. TG3 and 8, Trans Generation 1 and 8. (A) Tumor volumes. (B) Growth rate of tumors (vs. volume on Day 0). *, ** *P* <0.05, 0.01 vs. control. Bar=S.D.

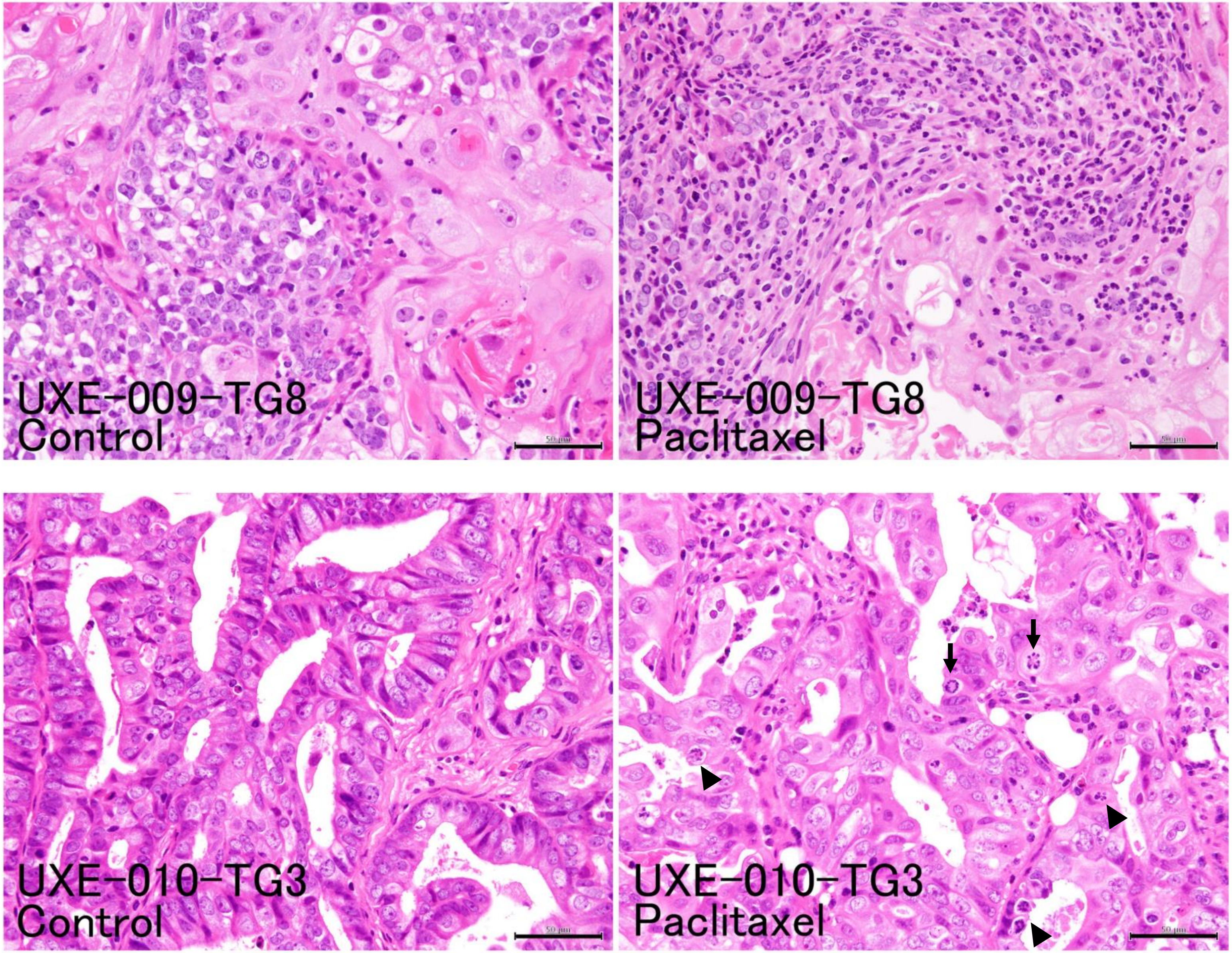

Supplementary Figure S6

Upper panels; apoptotic tumor cells are obvious in a paclitaxel-treated UXE-009-PDX.
Lower panels; tumor cell with apoptotic (arrowheads) and mitotic arrest (arrows) findings are scattered in a paclitaxel-treated UXE-010-PDX. Bar=50 μm.
